# Two Chloroflexi classes independently evolved the ability to persist on atmospheric hydrogen and carbon monoxide

**DOI:** 10.1038/s41396-019-0393-0

**Published:** 2019-03-14

**Authors:** Zahra F. Islam, Paul R. F. Cordero, Joanna Feng, Ya-Jou Chen, Sean K. Bay, Thanavit Jirapanjawat, Roslyn M. Gleadow, Carlo R. Carere, Matthew B. Stott, Eleonora Chiri, Chris Greening

**Affiliations:** 1 School of Biological Sciences, Monash University, Clayton, VIC 3800 Australia; 2 Department of Chemical and Process Engineering, University of Canterbury, Christchurch, 8041 New Zealand; 3 School of Biological Sciences, University of Canterbury, Christchurch, 8041 New Zealand

**Keywords:** Environmental microbiology, Microbial ecology

## Abstract

Most aerobic bacteria exist in dormant states within natural environments. In these states, they endure adverse environmental conditions such as nutrient starvation by decreasing metabolic expenditure and using alternative energy sources. In this study, we investigated the energy sources that support persistence of two aerobic thermophilic strains of the environmentally widespread but understudied phylum Chloroflexi. A transcriptome study revealed that *Thermomicrobium roseum* (class Chloroflexia) extensively remodels its respiratory chain upon entry into stationary phase due to nutrient limitation. Whereas primary dehydrogenases associated with heterotrophic respiration were downregulated, putative operons encoding enzymes involved in molecular hydrogen (H_2_), carbon monoxide (CO), and sulfur compound oxidation were significantly upregulated. Gas chromatography and microsensor experiments showed that *T. roseum* aerobically respires H_2_ and CO at a range of environmentally relevant concentrations to sub-atmospheric levels. Phylogenetic analysis suggests that the hydrogenases and carbon monoxide dehydrogenases mediating these processes are widely distributed in Chloroflexi genomes and have probably been horizontally acquired on more than one occasion. Consistently, we confirmed that the sporulating isolate *Thermogemmatispora* sp. T81 (class Ktedonobacteria) also oxidises atmospheric H_2_ and CO during persistence, though further studies are required to determine if these findings extend to mesophilic strains. This study provides axenic culture evidence that atmospheric CO supports bacterial persistence and reports the third phylum, following Actinobacteria and Acidobacteria, to be experimentally shown to mediate the biogeochemically and ecologically important process of atmospheric H_2_ oxidation. This adds to the growing body of evidence that atmospheric trace gases are dependable energy sources for bacterial persistence.

## Introduction

Bacteria from the phylum Chloroflexi are widespread and abundant in free-living microbial communities [1–4]. One reason for their success is their metabolic diversity; cultured strains from the phylum include heterotrophs, lithotrophs, and phototrophs adapted to both oxic and anoxic environments [5]. Cultured representatives of the phylum are classified into four classes by the genome taxonomy database [6], the primarily aerobic Chloroflexia and Ktedonobacteria and the anaerobic Anaerolineae and Dehalococcoidia [5]. Studies have provided insight into the metabolic strategies that anaerobic classes within the phylum use to adapt to oligotrophic niches [7, 8]. However, surprisingly little is known about how aerobic heterotrophic Chloroflexi colonise oxic environments. Global surveys have reported that Chloroflexi comprise 4.3% of soil bacteria [2] and 3.2% of marine bacteria [3]. However, the most dominant lineages within these ecosystems (notably Ellin6529 and SAR202) have not been cultivated [2, 6, 9]. Instead, most of our knowledge about the ecophysiological strategies of aerobic heterotrophic Chloroflexi is derived from studies on thermophilic isolates. Various strains from the classes Chloroflexia and Ktedonobacteria have been isolated and characterised from hot springs and geothermal soils [10–14].

Within geothermal environments, Chloroflexi strains are likely to encounter temporal and spatial variations in the availability of organic carbon compounds and other nutrients [15]. It is currently unknown how members of this phylum stay energised in response to these environmental perturbations. Carbon monoxide (CO) and molecular hydrogen (H_2_) of both geothermal and atmospheric origin are available in such environments and may be particularly important energy sources for sustaining growth and persistence [16–20]. Consistently, genomic, and metagenomic studies have revealed that Chloroflexi encode carbon monoxide dehydrogenases [21–23] and hydrogenases [21, 24–26] known to mediate aerobic respiration of these gases. Chloroflexi isolates have been shown to aerobically oxidise CO at a range of environmentally significant concentrations: *Thermomicrobium roseum* consumes high concentrations of CO when available during growth [21] and multiple *Thermogemmatispora* isolates have been shown to oxidise CO, including *T. carboxidovorans* to atmospheric concentrations (0.10 ppmv) [12]. While H_2_ oxidation has yet to be reported in aerobic heterotrophic Chloroflexi, strains of the phylum are known to encode the high-affinity group 1h [NiFe]-hydrogenase [24, 27]. This enzyme class has been shown to support bacterial persistence by mediating oxidation of atmospheric H_2_ (0.53 ppmv) [26, 28–36]. To date, atmospheric H_2_ oxidation has only been experimentally confirmed in Actinobacteria [28, 30–32, 34, 37] and two acidobacterial isolates [33, 38].

In this study, we investigated the persistence strategies of thermophilic isolates from two classes of Chloroflexi. We focused primarily on *Thermomicrobium roseum* (class Chloroflexia, formerly class Thermomicrobia [6]), a strain originally isolated from Toadstool Spring of Yellowstone National Park, USA [10]. This obligately aerobic bacterium is known to grow heterotrophically on a variety of carbohydrates, organic acids, and proteinaceous substrates [10, 13, 21]. Previous analyses have shown *T. roseum* encodes a type I carbon monoxide dehydrogenase and a group 1h [NiFe]-hydrogenase [21, 24], and can oxidise high concentrations of CO. However, the bacterium appears to be incapable of supporting chemolithoautotrophic growth and lacks key enzymes for the Calvin-Benson cycle [14, 21]. A combination of transcriptome sequencing and targeted activity assays were used to holistically determine the metabolic basis of persistence in this organism, including demonstrating that CO and H_2_ are oxidised by this strain during nutrient limitation. To help generalise these findings, we also investigated *Thermogemmatispora* sp. T81 (class Ktedonobacteria), a cellulolytic thermophilic strain which we previously isolated from geothermal soils in Tikitere, New Zealand [11, 39, 40]. Collectively, our results demonstrate that atmospheric H_2_ and CO serve as important energy sources that support the persistence of members of this phylum.

## Materials and methods

### Bacterial strains

*Thermomicrobium roseum* DSM 5159 [10, 13] and *Thermogemmatispora* sp. T81 [11, 39] were imported from the Extremophiles Research Group (GNS Science, Wairakei, New Zealand) culture collection in February 2017. Cultures of both bacterial isolates were routinely maintained in 120 mL serum vials sealed with treated lab-grade butyl rubber stoppers. Cultures of *T. roseum* contained 30 mL Castenholz media [41] supplemented with 1 g L^−1^ yeast extract and 1 g L^−1^ tryptone, whereas *Thermogemmatispora* sp. T81 cultures were maintained in 30 mL 10% R2A media [42]. Unless otherwise stated, both strains were incubated at 60 °C at an agitation speed of 150 rpm in an Eppendorf 40 Incubator. For *T. roseum*, cultures were inoculated to an OD_600_ of 0.03 and cells entered stationary phase within 48 h (OD_max_ = 0.75 to 1.0); growth curves confirmed that the strain entered stationary phase as a result of nutrient limitation, likely because of exhausting organic carbon supplies **(**Figure S1**)**. For *Thermogemmatispora* sp. T81, 1 mL of stationary-phase cells were inoculated into 29 mL medium and cultures were incubated for 294 h before gas consumption assays were performed. Sporulation of *Thermogemmatispora* sp. T81 at this timepoint was verified by light microscopy of Gram-stained cultures. Gram staining and 16S rRNA gene amplicon sequencing also confirmed that both cultures were axenic.

### Transcriptomics

Full transcriptome sequencing (RNA-Seq) was used to compare gene expression in *T. roseum* cultures under nutrient-rich (exponential phase; 10 mL, OD_600_ of 0.3) and nutrient-exhausted (stationary phase; 10 mL, OD_600_ of 0.75, 48 h post OD_max_) conditions. Biological triplicate samples for each condition were harvested by centrifugation (21,000 × *g*, 15 min, 4 °C), the supernatants were removed, and the cell pellets were resuspended in 1 mL of RNAlater Stabilisation Solution (ThermoFisher Scientific) prior to freezing at −20 °C. Extraction and sequencing of RNA was performed by Macrogen Inc., Seoul, Korea. Briefly, RNA was extracted using the RNeasy Plant Mini Kit (Qiagen), libraries were constructed using a TruSeq RNA v2 Sample Prep Kit (Illumina), and rRNA was removed using the Ribo-Zero rRNA Removal Kit (Illumina). The resultant complementary DNA was sequenced on an Illumina HiSeq4000 platform using a paired-end, 100 bp high-throughput protocol. Sequence analysis was performed using the automated cloud-based transcriptomics pipeline, AIR (Sequentia Biotech). Briefly, the steps performed were read trimming, read quality analysis using FastQC [43], and read mapping against the *T. roseum* reference genome (NCBI ID: NC_011959.1 [21]) using an intrinsic platform read aligner with default parameters. Aligned reads were checked for quality prior to data normalisation using the trimmed mean of M-values method [44] and the ‘normalizaData’ command within the R package HTSFilter [45]. A principle component analysis was then performed on the normalised data prior to statistical analysis using edgeR [46] to obtain differential gene expression counts.

### Gas chromatography

Gas chromatography measurements were performed to determine whether the two Chloroflexi strains could use atmospheric levels of CO and H_2_. Briefly, sealed serum vials containing stationary-phase cultures of *T. roseum* (72 hr post OD_max_ / 120 h post-inoculation) and sporulating cultures of *Thermogemmatispora* sp. T81 (294 h post-inoculation) were opened, equilibrated with ambient air (1 h), and resealed. These vials were then amended with H_2_ (*via* 1% v/v H_2_ in N_2_ gas cylinder, 99.999% pure) or CO (*via* 1% v/v CO in N_2_ gas cylinder, 99.999% pure) to achieve headspace concentrations of ~14 ppmv for each gas. The first headspace samples were collected within minutes after closure in order to measure the initial gas concentrations. The vials were maintained at the growth temperature (60 °C) and agitated (150 rpm) for the entire incubation period (75 h) to enhance H_2_, CO, and O_2_ transfer to the cultures. Six to nine headspace samples (1 mL) were collected at different time intervals using a gas-tight syringe to measure H_2_ and CO. Concomitantly, headspace gas concentrations in heat-killed negative controls (autoclaved; 30 mL) were measured to confirm that observed rates of gas consumption occurred due to a biotic process. Headspace gas concentrations were determined by gas chromatography using a pulsed discharge helium ionization detector [47, 48]. This customized trace gas analyser (model TGA-6791-W-4U-2, Valco Instruments Company Inc.) is designed to analyse a suite of atmospheric gases across six orders of magnitude of concentrations. Briefly, the system is configured to use two valves as injectors/backflushers, and two valves to front flush or heart cut from the precolumns (Mole Sieve 5A, set at 140 °C). Gases are then separated on the main columns (5′ X 1/8” HayseSep Db, set at 55 °C). The fifth valve is used as a sample loop selector to accommodate a larger range of gas concentrations. Concentrations of H_2_ and CO in each sample were regularly calibrated against ultra-pure H_2_ and CO gas standards of known concentrations. With the standards used, the limit of detection was 42 ppbv H_2_ and 9 ppbv CO.

### Kinetic measurements

The whole-cell kinetic parameters of H_2_ and CO oxidation in *T. roseum* were measured by comparing rates of gas consumption at different substrate concentrations. Briefly, the headspace of stationary-phase cultures (72 h post OD_max_) were amended with 100, 1000, or 4000 ppmv H_2_ or CO; whereas 100 ppmv mixing ratios were attained as described above, 100% H_2_ and 100% CO cylinders (99.999% pure) were used to attain mixing ratios of 1000 and 4000 ppmv. Cultures were incubated at 60 °C at an agitation speed of 300 rpm in an Eppendorf 40 Incubator. Headspace gas samples were measured at various time intervals (0, 0.5, 1, 2, 3, 4, and 5 h after substrate addition) by gas chromatography as described above. Reaction velocity relative to the gas concentration was measured at each timepoint and plotted on a Michaelis–Menten graph. Curves of best fit, *V*_max app_ values, and *K*_m app_ values were initially calculated in GraphPad Prism (version 7.01) using non-linear regression models (enzyme kinetics – substrate vs. velocity, Michaelis–Menten, least squares fit). Michaelis–Menten parameters were also derived using linear regressions based on Lineweaver-Burk, Eadie-Hofstee, and Hanes-Woolf plots [49].

### Activity staining

Hydrogenase and carbon monoxide dehydrogenase activity was stained using whole-cell lysates of stationary-phase cultures of *T. roseum*. Five hundred mL of culture was harvested by centrifugation (10,000 × *g*, 10 min, 4 °C), washed in phosphate-buffered saline solution (PBS; 137 mM NaCl, 2.7 mM KCl, 10 mM Na_2_HPO_4_ and 2 mM KH_2_PO_4_, pH 7.4), and resuspended in 16 mL lysis buffer (50 mM Tris-Cl, pH 8.0, 1 mM PMSF, 2 mM MgCl_2_, 5 mg ml^−1^ lysozyme, 40 µg ml^−1^ DNase, 10% glycerol). The resultant suspension was then lysed by passage through a Constant Systems cell disruptor (40,000 psi, four times), with unbroken cells removed by centrifugation (10,000 × *g*, 20 min, 4 °C). Protein concentration was calculated using the bicinchoninic acid assay [50] against bovine serum albumin standards. Next, 20 µg protein was loaded onto a native 7.5% (w/v) Bis-Tris polyacrylamide gel prepared as described elsewhere [51] and run alongside a protein standard (NativeMark Unstained Protein Standard, Thermo Fisher Scientific) at 25 mA for 1.5 h. The gel was cut into three sections that were stained either for total protein, hydrogenase activity, or carbon monoxide dehydrogenase activity. For total protein staining, the gel section was incubated in AcquaStain Protein Gel Stain (Bulldog Bio) at room temperature for 3 h. For hydrogenase staining [28], the gel section was incubated in 50 mM potassium phosphate buffer (pH 7.0) supplemented with 500 µM nitroblue tetrazolium chloride (NBT) in an anaerobic jar (5% H_2_, 10% CO_2_, 85% N_2_ v/v) at 60 °C for 1 h. For carbon monoxide dehydrogenase staining [52], the gel section was incubated in 50 mM Tris-HCl buffer (pH 7.5) containing 50 µM NBT and 100 µM phenazine methosulfate in an anaerobic jar (100% CO v/v atmosphere) maintained at 60 °C for 1 h.

### Electrode measurements

For *T. roseum* cultures, rates of H_2_ oxidation with and without treatment of respiratory chain uncouplers were measured amperometrically, following previously established protocols [53, 54]. Prior to the start of measurement, a Unisense H_2_ microsensor electrode was polarised at +800 mV for 1 h using a Unisense multimeter and calibrated with standards of known H_2_ concentration. Gas-saturated PBS was prepared by bubbling the solution with 100% (v/v) of either H_2_ or O_2_ for 5 min. For untreated cells, 1.1 mL microrespiration assay chambers were sequentially amended with stationary-phase *T. roseum* cell suspensions (OD_600_ = 1; 0.9 mL), H_2_-saturated PBS (0.1 mL), and O_2_-saturated PBS (0.1 mL) stirred at 250 rpm, 37 °C. Following measurements of untreated cells, the assay mixtures were treated with 100 µM carbonyl cyanide *m*-chlorophenyl hydrazine (CCCP), 10 µM nigericin, or 10 µM valinomycin. Changes in H_2_ concentrations were recorded using Unisense Logger Software. Upon observing a linear change in H_2_ concentration, initial rates of consumption were calculated over a period of 20 s and normalised against total protein concentration.

### Phylogenetic analyses

Phylogenetic trees were constructed to investigate the evolutionary history and distribution of uptake hydrogenases and carbon monoxide dehydrogenases within the Chloroflexi phylum. Specifically, the catalytic subunits of [NiFe]-hydrogenases (HhyL and homologues) and type I carbon monoxide dehydrogenases (CoxL) were retrieved from Chloroflexi genomes and metagenome-assembled genomes (MAGs) in the NCBI RefSeq database via protein BLAST [55] in October 2018. Using MEGA7 [56], the amino acid sequences were aligned with reference sequences [23, 24] with ClustalW and evolutionary relationships were visualised by constructing a maximum-likelihood phylogenetic tree; specifically, initial trees for the heuristic search were obtained automatically by applying Neighbour-Join and BioNJ algorithms to a matrix of pairwise distances estimated using a JTT model, and then selecting the topology with superior log likelihood value. Gaps were treated with partial deletion and trees were bootstrapped with 100 replicates.

## Results and discussion

### *Thermomicrobium roseum* upregulates hydrogenase and carbon monoxide dehydrogenase expression during a coordinated response to nutrient starvation

**Fig. 1:**
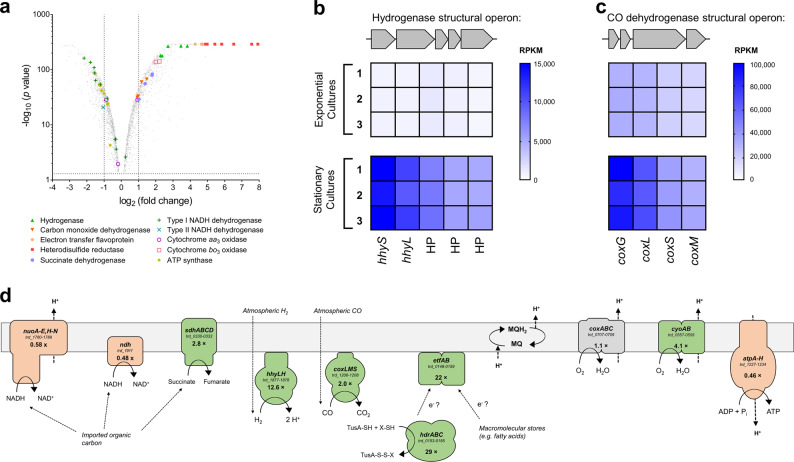
Differential gene expression of nutrient-rich (exponential phase) and nutrient-limited (stationary phase) cultures of *Thermomicrobium roseum*. **a** Volcano plot showing relative expression change of genes following nutrient limitation. The fold-change shows the ratio of normalised transcript abundance of three stationary phase cultures divided by three exponential phase cultures (biological replicates). Each gene is represented by a grey dot and respiratory genes are highlighted as per the legend. **b,** **c** Heat maps of normalised abundance of the putative operons encoding the structural subunits of the group 1h [NiFe]-hydrogenase (*hhyLS*; **b**) and type I carbon monoxide dehydrogenase (*coxLSM*; **c**). The read counts per kilobase million (RPKM) are shown for three exponentially growing and three stationary phase biological replicates. HP = hypothetical protein. **d** Differential regulation of the respiratory complexes mediating aerobic respiration of organic and inorganic compounds. Complexes are differentially shaded depending on whether they are significantly upregulated (green), downregulated (orange), or unchanged (grey) in nutrient-limited compared to nutrient-rich cultures. Gene names, loci numbers, and average fold changes in transcriptome abundance are shown for each complex. Shown are the structural subunits of type I NADH dehydrogenase (*nuoA-E,H-N*), type II NADH dehydrogenase (*ndh*), succinate dehydrogenase (*sdhA-D*), group 1h [NiFe]-hydrogenase (*hhyLS*), type I carbon monoxide dehydrogenase (*coxLMS*), heterodisulfide reductase (*hdrABC*), electron transfer flavoprotein (*etfAB*), sulfur-carrier protein (*tusA*), cytochrome *aa*_3_ oxidase (*coxABC*), cytochrome *bo*_3_ oxidase (*cyoAB*), and ATP synthase (*atpA-H*). Note that the physiological role of the highly upregulated *hdrABC*, *etfAB*, and *tusA* genes is yet to be experimentally validated in *T. roseum*

We compared the transcriptomes of triplicate *T. roseum* cultures under nutrient-rich (exponential growing) and nutrient-limited (stationary phase) conditions. A total of 401 genes were significantly upregulated and 539 genes were significantly downregulated by at least two-fold (*p* < 10^−6^) in response to nutrient limitation **(**Fig. 1a; Table S1**)**. Three major trends were observed with respect to energy acquisition and utilisation. Firstly, genes associated with energetically expensive processes were downregulated, including those encoding ribosomal proteins, cytochrome *c* and menaquinone biosynthesis enzymes, and the megaplasmid-encoded chemotactic and flagellar apparatus **(**Table S1**)**. Secondly, there was evidence of mobilisation of internal carbon stores, including an acetoin dehydrogenase complex and an electron transfer flavoprotein complex (ETF). Thirdly, the expression profiles indicate there is extensive remodelling of the respiratory chain. Two primary respiratory dehydrogenases involved in heterotrophic growth (type I and II NADH dehydrogenases) were downregulated, whereas complexes involved in lithotrophic energy generation and a succinate dehydrogenase were upregulated **(**Fig. 1a; Table S1**)**. In both conditions, the terminal oxidases that mediate aerobic respiration were highly expressed and there was no evidence of the use of other electron acceptors; the cytochrome *aa*_3_ oxidase was expressed in both phases and the alternative cytochrome *bo*_3_ oxidase was upregulated during stationary phase. In contrast, the F_1_F_o_-ATPase (ATP synthase) was downregulated, a finding consistent with an expected decrease in the availability of respiratory electron donors during nutrient limitation **(**Table S1**)**.

*Thermomicrobium roseum* upregulates genes associated with H_2_ and CO metabolism under nutrient-limiting conditions. The genes encoding the structural subunits of a group 1h [NiFe]-hydrogenase (*hhyLS*; trd_1878–1877) [24, 25, 57], which are a class of oxygen-tolerant enzymes known to mediate atmospheric H_2_ oxidation [28, 31, 33, 58, 59], were upregulated by an average of 12.6-fold **(**Fig. 1b**)**. Also upregulated were the conserved hypothetical proteins *hhaABC* (trd_1876–1874; 5.5-fold) [27], encoded on the same putative operon as the structural subunits, as well as a separate putative operon of maturation factors (trd_1873–1863; 3.1-fold) **(**Figure S2; Table S1**)**. The structural (trd_1206–1208) and maturation (trd_1209–1215) subunits encoding a type I carbon monoxide dehydrogenase were upregulated by an average of two-fold **(**Fig. 1c & S2**)** in response to nutrient limitation. Consistent with previous reports of CO utilisation during growth in this organism [21], carbon monoxide dehydrogenase genes were highly expressed in both exponential and stationary-phase cultures. **(**Fig. 1c; Table S1**)**. This suggests that *T. roseum* uses CO to supplement available organic carbon during growth (mixotrophy) and persistence. These findings are broadly similar to observations made in other phyla, notably Actinobacteria and Proteobacteria, that hydrogenase and carbon monoxide dehydrogenase expression are induced by organic carbon limitation [28, 31, 60–64].

Overall, the greatest differential in gene expression involved a 19-gene cluster (trd_0160–0142) putatively involved with the oxidation of sulfur compounds. The cluster contains gene encoding a putative soluble heterodisulfide reductase (*hdrABC*), an electron transfer flavoprotein complex (*etfAB*), three sulfur-carrier proteins (*tusA*, *dsrE1*, *dsrE2*), three lipoate-binding proteins (*lbpA*), and various hypothetical proteins, which are upregulated by an average of 45-fold during persistence. Most of these components have homologues in a system recently shown to mediate the oxidation of diverse organic and inorganic sulfur compounds in *Hyphomicrobium denitrificans* [65, 66]. One role of this cluster may be to mediate the activation and oxidation of endogenous or exogenous thiol-containing compounds. To achieve this, we predict that the Hdr complex catalyses disulfide bond formation between the thiol compound and a sulfur-carrier protein (e.g., TusA); the Hdr complex then transfers the liberated electrons into the respiratory chain, possibly via the ETF complex. Supporting this notion, thiol oxidation to disulfide is exergonic with oxygen as the terminal electron acceptor. While Hdr complexes are best-characterized for their roles in heterodisulfide reduction in methanogenic archaea [67], they have also been studied in sulfur-oxidizing and sulfate-reducing bacteria, where they have been predicted to be physiologically reversible [68, 69]. Consistently, the Hdr complex of *T. roseum* is most closely related to those of sulfur-oxidising *Sulfobacillus*, *Hyphomicrobium*, and *Acidithiobacillus* strains [65, 70, 71]. It seems plausible that *T. roseum* would benefit from a survival advantage if it can harness reduced sulfur compounds available in geothermal springs. However, further work is needed to verify the activity, substrates, and physiological role of this system.

Collectively, these findings show that *T. roseum* is more metabolically flexible than previously thought. Fig. 1d illustrates the predicted remodelling of the respiratory chain that occurs during the transition from nutrient-rich to nutrient-limited conditions. The upregulation of enzymes involved in harnessing inorganic compounds, in conjunction with the downregulation of gene clusters involved in NADH oxidation, suggests that *T. roseum* has evolved mechanisms to maintain aerobic respiration despite nutrient fluctuations and deprivation within its environment.

### *T. roseum* aerobically oxidises H_2_ and CO at a wide range of concentrations, including sub-atmospheric levels, during persistence

**Fig. 2:**
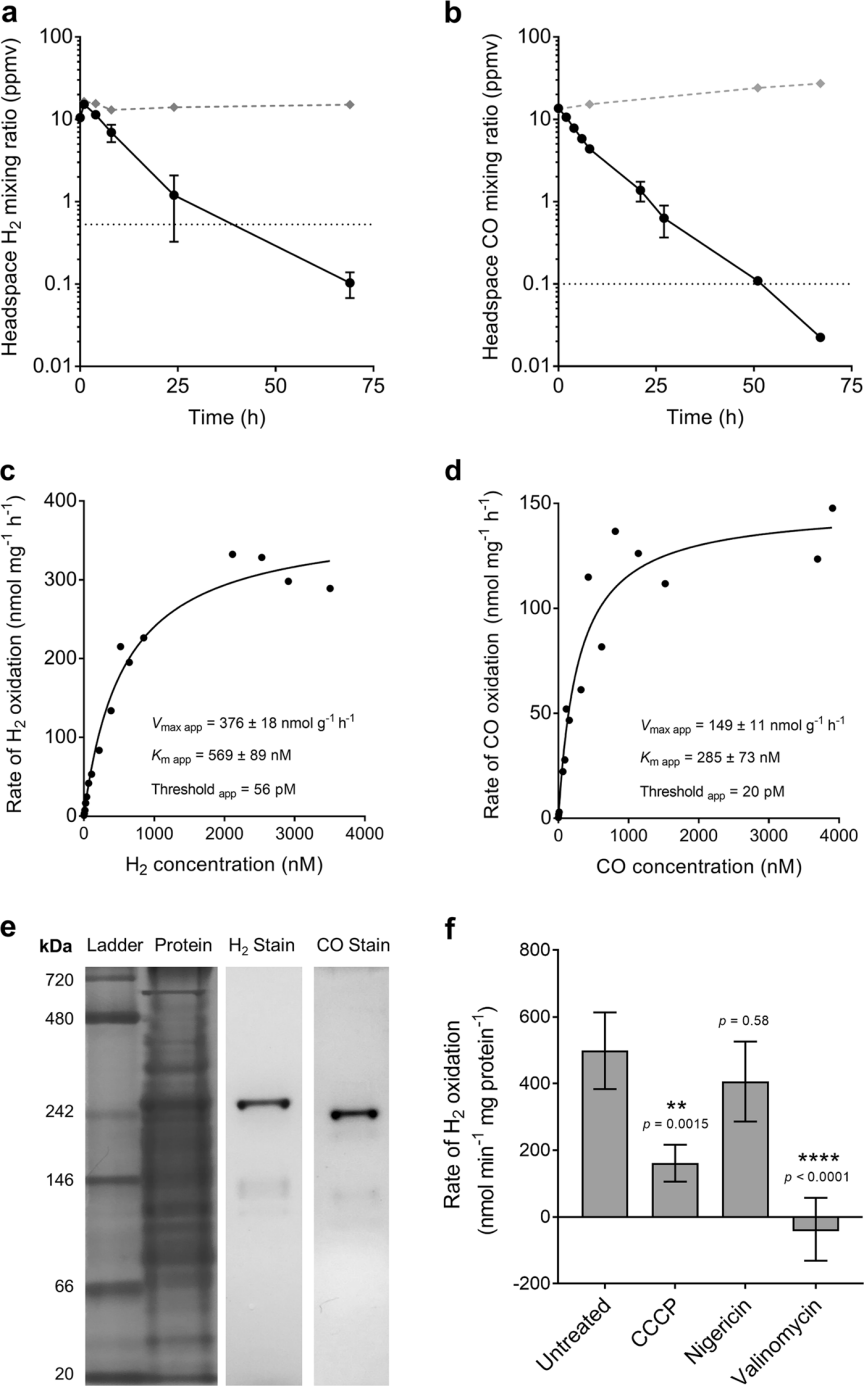
Hydrogenase and carbon monoxide dehydrogenase activity of *Thermomicrobium roseum* cultures during nutrient limitation. **a,** **b** Oxidation of molecular hydrogen (H_2_; **a**) and carbon monoxide (CO; **b**) to sub-atmospheric levels by *T. roseum* cultures. Error bars show standard deviations of three biological replicates, with heat-killed cells monitored as a negative control (grey dashed lines). Mixing ratios of H_2_ and CO are displayed on a logarithmic scale and dotted lines show the average atmospheric mixing ratios of H_2_ (0.53 ppmv) and CO (0.10 ppmv). **c,** **d** Apparent kinetic parameters of H_2_ (**c**) and CO (**d**) oxidation by *T. roseum* whole cells. Curves of best fit and kinetic parameters were calculated based on a Michaelis–Menten non-linear regression model. Values calculated based on Lineweaver-Burk, Hanes-Woolf, and Eadie-Hofstee plots are shown in Table S2. **e** Zymographic observation of hydrogenase and carbon monoxide dehydrogenase activity in *T. roseum* whole-cell lysates. The first two lanes show protein ladder and whole protein stained with Coomassie Blue. The third and fourth lanes show hydrogenase and carbon monoxide dehydrogenase activity stained with the artificial electron acceptor nitroblue tetrazolium in a H_2_-rich and CO-rich atmosphere respectively. **f** Amperometric measurements of hydrogenase activity in *T. roseum* whole cells. The rate of H_2_ oxidation was measured with a hydrogen electrode before and after treatment with the respiratory uncouplers and ionophores carbonyl cyanide *m*-chlorophenyl hydrazine (CCCP), nigericin, and valinomycin

The high expression levels for genes encoding the group 1h [NiFe]-hydrogenase and type I carbon monoxide dehydrogenase suggested that *T. roseum* may support persistence by oxidising atmospheric H_2_ and CO. To test this, we incubated nutrient-limited cultures of *T. roseum* in an ambient air headspace supplemented with ~14 ppmv of either H_2_ or CO and monitored their consumption using gas chromatography. In agreement with our hypothesis, cultures aerobically oxidised both gases in a first-order kinetic process; within 71 h, mixing ratios of these gases (103 ppbv H_2_, 22 ppbv CO) were five times below atmospheric levels **(**Fig. 2a, b**)**. This constitutes the first observation of both aerobic H_2_ respiration and atmospheric H_2_ oxidation within the phylum Chloroflexi.

Whole-cell kinetic measurements revealed that *T. roseum* efficiently oxidises H_2_ and CO across a wide range of concentrations through hydrogenase and carbon monoxide dehydrogenase activity. In cultures, the enzymes display a moderate apparent velocity (*V*_max app_ of 376 nmol H_2_ and 149 nmol CO g^−1^ of protein min^−1^) and moderate apparent affinity (*K*_m app_ of 569 nM H_2_ and 285 nM CO) for these substrates **(**Fig. 2c, d; Table S2**)**. With respect to carbon monoxide dehydrogenase, these observations are consistent with the organism being able to utilise CO at elevated concentrations for growth [21] and atmospheric concentrations for persistence. The apparent kinetic parameters of the group 1h [NiFe]-hydrogenase are more similar to those recently described for the verrucomicrobial methanotroph *Methylacidiphilum fumariolicum* (*K*_m_ = 600 nM) [72] than to the high-affinity, low-activity hydrogenases of previously described atmospheric H_2_ scavengers (*K*_m_ < 50 nM) [28, 31, 33]. Altogether, these findings suggest that *T. roseum* can take advantage of the elevated H_2_ and CO concentrations when available through geothermal activity and subsist on atmospheric concentrations of these gases otherwise.

Consistent with the observed whole-cell activities, cell-lysates run on native polyacrylamide gels strongly stained for hydrogenase and carbon monoxide dehydrogenase activity **(**Fig. 2e**)**. The molecular weight of the major bands were, respectively, at the expected molecular weight for a carbon monoxide dehydrogenase dimer [266 kDa, (CoxLMS)_2_] and slightly below the expected molecular weight of a hydrogenase dimer [210 kDa, (HhyLS)_2_]. This is compatible with biochemical studies in other organisms that have shown type I carbon monoxide dehydrogenases and group 1h [NiFe]-hydrogenases form homodimers [58, 59, 73]. We next verified that the hydrogenase was coupled to the respiratory chain by measuring H_2_ oxidation using a H_2_ electrode under aerobic conditions. Untreated cells oxidised H_2_ at a rapid rate. This activity decreased by 2.5-fold upon addition of the respiratory uncoupler CCCP and ceased upon addition of the ionophore valinomycin, whereas no significant change in H_2_ oxidation rate was observable with the protonophore nigericin **(**Fig. 2f**)**. The combination of these results suggests that the oxidation of hydrogen is tightly coupled to the respiratory chain and this interaction may be linked to the electrical gradient (Δψ), but not pH gradient (ΔpH), of the membrane.

Findings from the transcriptome analysis and activity studies therefore suggest that *T. roseum* persists through oxidation of atmospheric H_2_ and CO. We propose that the group 1h [NiFe]-hydrogenase and type I carbon monoxide dehydrogenase directly use electrons derived from atmospheric H_2_ and CO to support aerobic respiration **(**Fig. 1d). It is probable that these electrons are relayed via electron carriers into the menaquinone pool and are subsequently transferred to the terminal oxidases. However, further studies are needed to confirm how these proteins functionally and physically interact with the respiratory chain, including their localisation and which electron carriers they interact with. Due to the genetic intractability of Chloroflexi and the lack of specific hydrogenase or carbon monoxide dehydrogenase inhibitors, we were also unable to determine the necessity of either H_2_ or CO oxidation for prolonged survival for this organism. However, previous studies have demonstrated that genetic deletion of the group 1h [NiFe]-hydrogenase reduces longevity of *M. smegmatis* cells [29, 30, 60] and *Streptomyces avermitilis* exospores [31, 32].

### Scavenging of atmospheric gases is potentially a common persistence strategy within the aerobic heterotrophic Chloroflexi

Having demonstrated that *T. roseum* oxidises atmospheric trace gases during persistence, we subsequently investigated whether this is a common strategy employed by the Chloroflexi. We first analysed the respiratory capabilities of *Thermogemmatispora* sp. T81, a heterotrophic cellulolytic and sporulating thermophile, which we previously isolated from geothermal soils from Tikitere, New Zealand [11, 40]. Analysis of the organism’s genome (Assembly ID: GCA_003268475.1) indicated that it encodes core respiratory chain components similar to *T. roseum*, including primary dehydrogenases (*nuo*, *ndh*, *sdh*), terminal oxidases (*cox*, *cyo*), and ATP synthase (*atp*). The genome also encodes putative operons for the structural subunits of a group 1h [NiFe]-hydrogenase, the maturation factors of this hydrogenase, and structural subunits of a type I carbon monoxide dehydrogenase **(**Figure S3**)**. However, homologues of the putative heterodisulfide reductase and ETF complexes encoded by *T. roseum* are absent from the *Thermogemmatispora* sp. T81 genome.

**Fig. 3:**
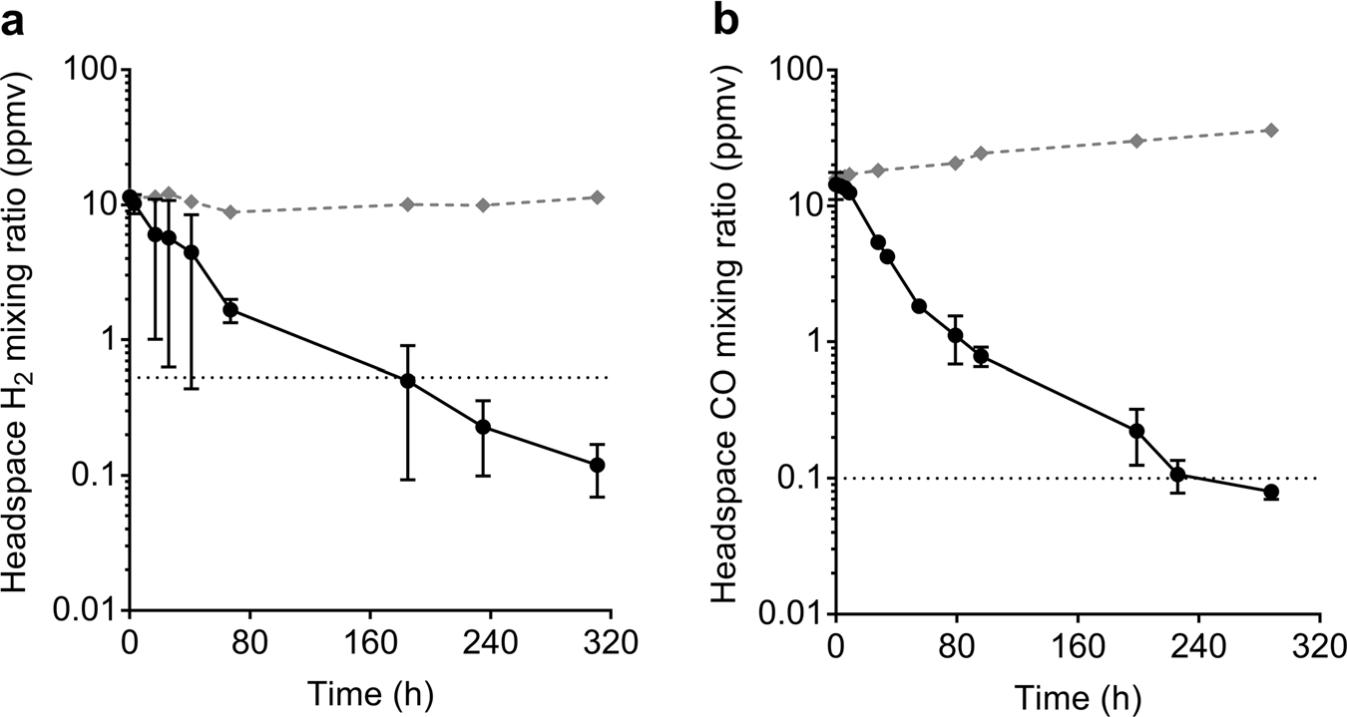
Hydrogenase and carbon monoxide dehydrogenase activity of *Thermogemmatispora* sp. T81 during sporulation. Oxidation of molecular hydrogen (H_2_; **a**) and carbon monoxide (CO; **b**) to sub-atmospheric levels by *Thermogemmatispora* sp. T81 cultures. Error bars show standard deviations of three biological replicates, with heat-killed cells monitored as a negative control (grey dashed lines). Mixing ratios of H_2_ and CO are displayed on a logarithmic scale and dotted lines show the average atmospheric mixing ratios of H_2_ (0.53 ppmv) and CO (0.10 ppmv)

We verified that sporulating cultures of *Thermogemmatispora* sp. T81 actively consume H_2_ and CO. The organism slowly oxidised available H_2_ and CO in the headspace to sub-atmospheric levels (120 ppbv H_2_, 70 ppbv CO) over ~320 h **(**Fig. 3a, b**)**. Although this strain has previously been shown to oxidise carbon monoxide [12], this is the first observation that it can do so to sub-atmospheric concentrations and during persistence. These results suggest that, despite their distinct evolutionary histories and ecological niches, *Thermogemmatispora* sp. T81 and *T. roseum* have both evolved similar metabolic strategies to survive nutrient limitation.

**Fig. 4:**
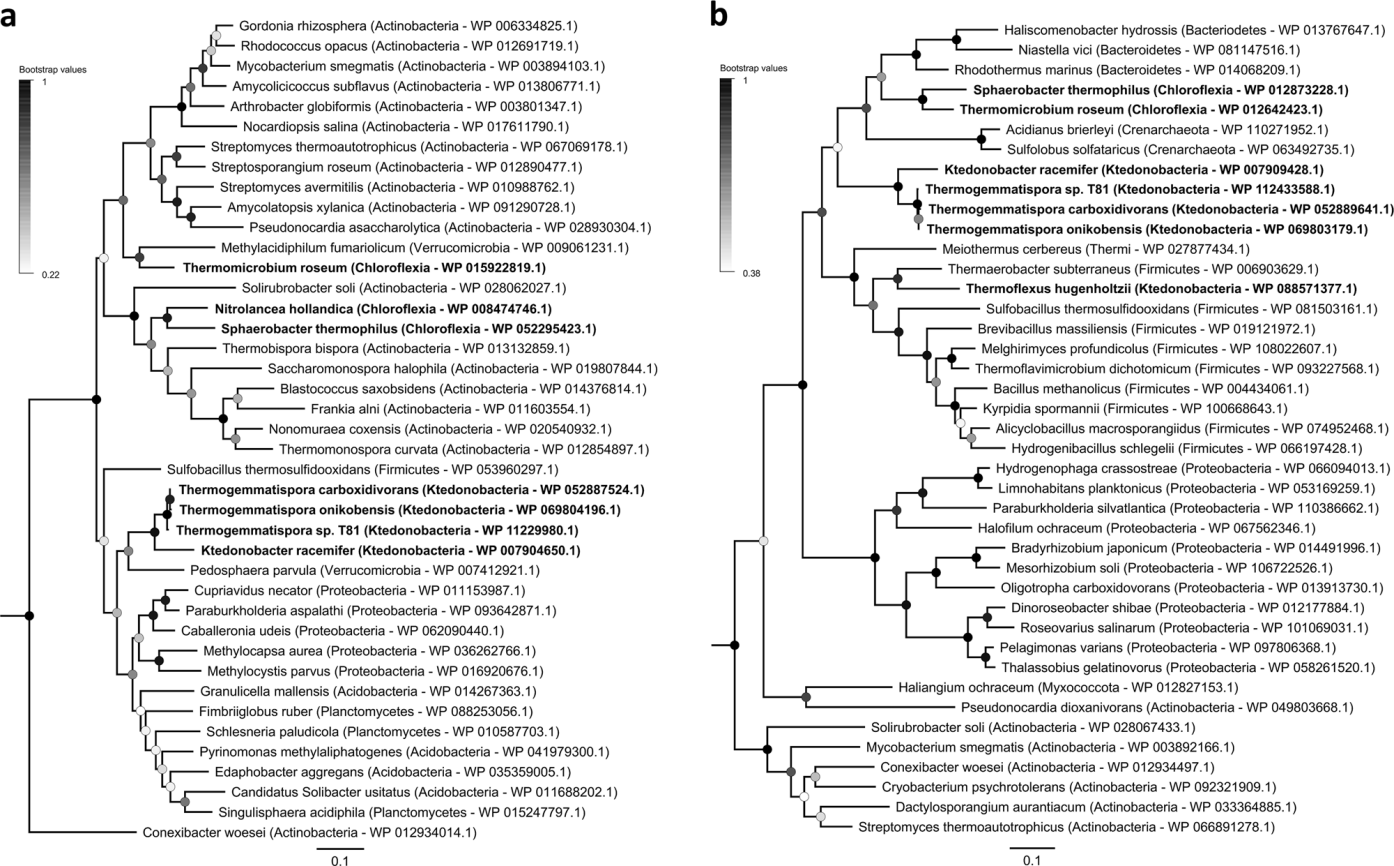
Evolutionary history of the group 1h [NiFe]-hydrogenase and type I carbon monoxide dehydrogenase. Phylogenetic trees showing the distribution and evolutionary history of the catalytic (large) subunits of the group 1h [NiFe]-hydrogenase (*hhyL*; **a**) and type I carbon monoxide dehydrogenase (*coxL*; **b**) in the phylum Chloroflexi. Chloroflexi sequences (labelled by class) are shown in bold against reference sequences (labelled by phylum). Trees were constructed using amino acid sequences through the maximum-likelihood method (gaps treated with partial deletion) and were bootstrapped with 100 replicates. The trees were respectively rooted with group 1g [NiFe]-hydrogenase sequences (WP_011761956.1, WP_048100713.1) and type II carbon monoxide dehydrogenase sequences (WP_011388721.1, WP_012893108.1). The distribution of other respiratory uptake hydrogenases within genomes and metagenome-assembled genomes (MAGs) in the phylum Chloroflexi is shown in Figure S4. The distribution of type I carbon monoxide dehydrogenases within metagenome-assembled genomes (MAGs) in the phylum Chloroflexi is shown in Figure S5

Analysis of the distribution of hydrogenases and carbon monoxide dehydrogenases within publicly available reference genomes showed that genetic capacity for trace gas scavenging is a common trait among aerobic Chloroflexi. Specifically, group 1h [NiFe]-hydrogenases and type I carbon monoxide dehydrogenases were encoded in three of the four reference genomes within the Thermomicrobiales (class Chloroflexia) and four of the five reference genomes within the Ktedonobacteriales (class Ktedonobacteria) **(**Fig. 4a, b**)**. The latter includes the genomes of the heterotrophic soil bacterium *Ktedonobacter racemifer* [74] and the nitrite-oxidising bioreactor isolate *Nitrolancea hollandica* [75]. In addition, seven strains within the photosynthetic order Chloroflexales encoded group 1f and/or group 2a [NiFe]-hydrogenases **(**Figure S4**)**. These hydrogenase classes have been shown to mediate aerobic H_2_ oxidation in a range of bacteria, including to sub-atmospheric concentrations in *Acidobacterium ailaaui* and *M. smegmatis* respectively [28, 38]. Moreover, a metatranscriptome study revealed that homologs of the group 1f [NiFe]-hydrogenase of *Roseiflexus* species are highly expressed in geothermal microbial mats at night [76]. Hence, it is likely that the traits of aerobic H_2_ respiration and possibly atmospheric H_2_ oxidation extends to the photosynthetic strains of this phylum. A range of metagenome-assembled genomes, including from the abundant candidate class Ellin6529 [2, 26], also encoded genes for aerobic H_2_ and CO oxidation **(**Figure S4 & S5**)**. Consistent with previous reports, Dehalococcoidia encode group 1a [NiFe]-hydrogenases known to facilitate dehalorespiration [77–79].

Our analyses suggest that the capacity for atmospheric H_2_ and CO oxidation may have evolved on two or more occasions within the Chloroflexi. Phylogenetic trees show that the group 1h [NiFe]-hydrogenases from Chloroflexia and Ktedonobacteria are divergent and fall into two distinct, robustly supported branches **(**Fig. 4a**)**. It is therefore more likely that Chloroflexia and Ktedonobacteria independently acquired these enzymes, for example as a result of horizontal gene transfer events from other Terrabacteria, rather than vertically inheriting them from a common ancestor. Phylogenetic analysis also suggests that the type I carbon monoxide dehydrogenase may have also been acquired on two or three occasions in this phylum **(**Fig. 4b**)**. In line with their probable independent acquisition, the putative operons encoding the hydrogenase and carbon monoxide dehydrogenase in *T. roseum* **(**Figure S2) and *Thermogemmatispora* sp. T81 **(**Figure S3) are distinctly organized. For example, the structural and accessory factors of carbon monoxide dehydrogenase are encoded in a single putative operon in *Thermogemmatispora* sp. T81 (*coxMSLIG*), but are separated into a structural operon (*coxGSLM*) and accessory operon (including *coxG* and *coxE*) in *T. roseum*. These findings agree with previous inferences of horizontal dissemination of *hhyL* and *coxL* genes [23, 24, 27] and suggest there is strong selective pressure for the acquisition of metabolic enzymes that support persistence. However, other explanations for their observations cannot be ruled out and further analysis is required to unravel the complex evolutionary histories of hydrogenases and carbon monoxide dehydrogenases.

### Ecological and biogeochemical significance of metabolic flexibility and trace gas oxidation in Chloroflexi

Aerobic heterotrophic bacteria from the phylum Chloroflexi are more metabolically versatile than previously thought. The transcriptome analyses clearly show that *T. roseum* regulates its metabolism in response to nutrient limitation, enabling persistence on a combination of exogenous inorganic compounds and likely endogenous carbon reserves. In support of this, gas chromatography measurements showed that the bacterium efficiently oxidises H_2_ and CO down to sub-atmospheric concentrations during persistence through an aerobic respiratory process. We made similar findings for the ktedonobacterial isolate *Thermogemmatispora* sp. T81, suggesting that trace gas scavenging might be a common persistence strategy employed by aerobic Chloroflexi. Analyses of primary sequence phylogeny and operon structure indicate that the group 1 h [NiFe]-hydrogenases and carbon monoxide dehydrogenases within these organisms fall into different clades and are relatively divergent. Hence, it is probable that these organisms have horizontally acquired the capacity to oxidise atmospheric H_2_ and CO via separate events, though other explanations are possible. The apparent convergence in persistence strategies is notable given the distinct evolutionary histories, persistence morphologies (i.e., sporulation in T81), and ecological niches of these bacteria. Resource generalism is therefore likely to be a common ecological strategy for the survival of Chloroflexi in environments where organic carbon and other nutrients may be periodically scarce.

More broadly, these findings provides pure culture support for the hypothesis that atmospheric carbon monoxide serves as an energy source for persistence [26]. Our findings suggest that the expression and activity of carbon monoxide dehydrogenase is linked to persistence, and provide evidence that atmospheric CO may serve as an electron donor for the aerobic respiratory chain in this condition. Indeed, as with atmospheric H_2_, atmospheric CO is likely to be a dependable energy source for microbial survival given its ubiquity, diffusibility, and energy density. Integrating these findings with the wider literature, it is probable that atmospheric CO oxidation is a general strategy supporting long-term survival of aerobic heterotrophic bacteria. Indeed, various heterotrophic bacteria have previously been inferred to be capable of oxidising atmospheric CO, including Proteobacteria [80–83], Actinobacteria [84, 85], and a *Thermogemmatispora* strain [12]. Moreover, other datasets have shown that carbon monoxide dehydrogenase expression is activated during nutrient limitation in other aerobic organisms [60–64]. However, in contrast to atmospheric H_2_ [29, 32], it remains to be validated through genetic and biochemical studies that atmospheric CO oxidation can enhance survival of bacteria during persistence. In line with previous activity-based measurements [21], the transcriptome analysis shows that *T. roseum* expresses carbon monoxide dehydrogenase at high levels during growth. Unlike carboxydotrophs such as *Oligotropha carboxidovorans* [62, 73, 86], *T. roseum* as a carboxydovore cannot grow chemolithoautotrophically [14, 21] and instead appears to use CO as an additional energy source during heterotrophic growth. The wide kinetic range of the *T. roseum* carbon monoxide dehydrogenase in whole cells likely enables this isolate to both persist on ubiquitously available atmospheric CO [87] and grow mixotrophically in microenvironments where CO is available at elevated concentrations (up to 6000 ppmv) through geothermal activity [17].

Finally, this study establishes Chloroflexi as the third phylum experimentally shown to scavenge atmospheric H_2_, following the Actinobacteria [28, 31, 34, 37, 57] and Acidobacteria [33, 38]. The findings made here are similar to those previously reported for the actinobacterium *Mycobacterium smegmatis* [28, 60] and acidobacterium *Pyrinomonas methylaliphatogenes* [33], both of which also shift from heterotrophic respiration to atmospheric H_2_ oxidation in response to energy limitation, including through expressing group 1h [NiFe]-hydrogenases. Given at least four other cultured phyla **(**Fig. 4a**)** and two candidate phyla [26] also encode group 1h [NiFe]-hydrogenases, it seems increasingly likely that atmospheric H_2_ serves as a general energy source for aerobic heterotrophic bacteria. This observation is also potentially biogeochemically significant, given aerobic soil bacteria are known to be the main sink in the global hydrogen cycle [88]. Further work, however, is needed to test these whether these principles extend to the still enigmatic Chloroflexi species inhabiting mesophilic soil environments.

## Supplementary information

Supplemental material

Table S1
