# Supplementary material for: Two Chloroflexi classes independently evolved the ability to persist on atmospheric hydrogen and carbon monoxide
Source: ISME J. 2019 Mar 14;13(7):1801–13. doi: 10.1038/s41396-019-0393-0 (PMC6776052; doi:10.1038/s41396-019-0393-0)

Supplementary Information

Table S1 (xlsx). Transcript read counts for RNA-seq experiment comparing gene expression of three exponential phase (nutrient-rich) and three stationary phase (nutrient-limited) cultures of *Thermomicrobium roseum*.

Table S2. Comparison of four methods to determine apparent kinetic parameters for H_2 and CO oxidation for whole cells of *Thermomicrobium roseum*.

**Method**	$V_{max\ app}$ H_2	$K_{m\ app}$ H_2	$V_{max\ app}$ CO	$K_{m\ app}$ CO
Nonlinear regression	376	569	149	285
Lineweaver-Burk plot	319	688	175	427
Hanes-Woolf plot	372	624	133	220
Eadie-Hofstee plot	377	639	137	244
Average	361	630	149	294

Figure S1. Growth curve of *Thermomicrobium roseum* DSM 5159. Cultures were grown in 120 mL serum vials containing 30 mL Castenholz media supplemented with 1 g L^{-1} yeast extract and 1 g L^{-1} tryptone. They were inoculated at a starting OD_{600} of 0.03 and incubated in an orbtial shaker at 150 rpm, 60°C. After cells entered stationary-phase, at 72 h the cultures were either supplemented with 1 g L^{-1} tryptone, reaerated for 1 h, or left untreated. This confirmed that cultures entered stationary-phase due to nutrient-limitation rather than oxygen-limitation.

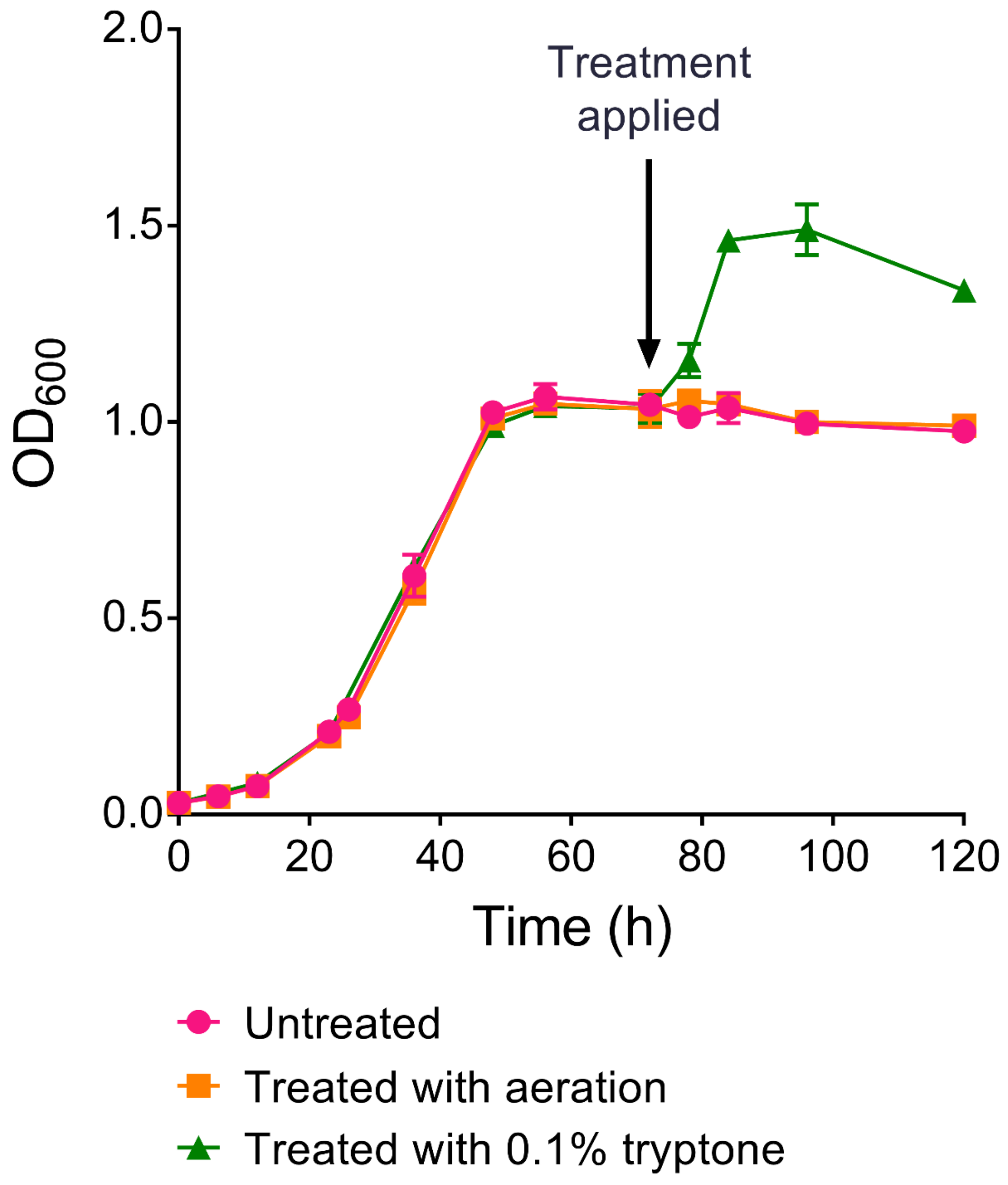

Figure S2. Structure of the putative operons encoding the group 1h [NiFe]-hydrogenase, type I carbon monoxide dehydrogenase, and heterodisulfide reductase / electron transfer flavoprotein complex in *Thermomicrobium roseum*. Genes are differentially coloured depending on whether they encode structural subunits (green), accessory and maturation factors (yellow), or hypothetical proteins (grey).

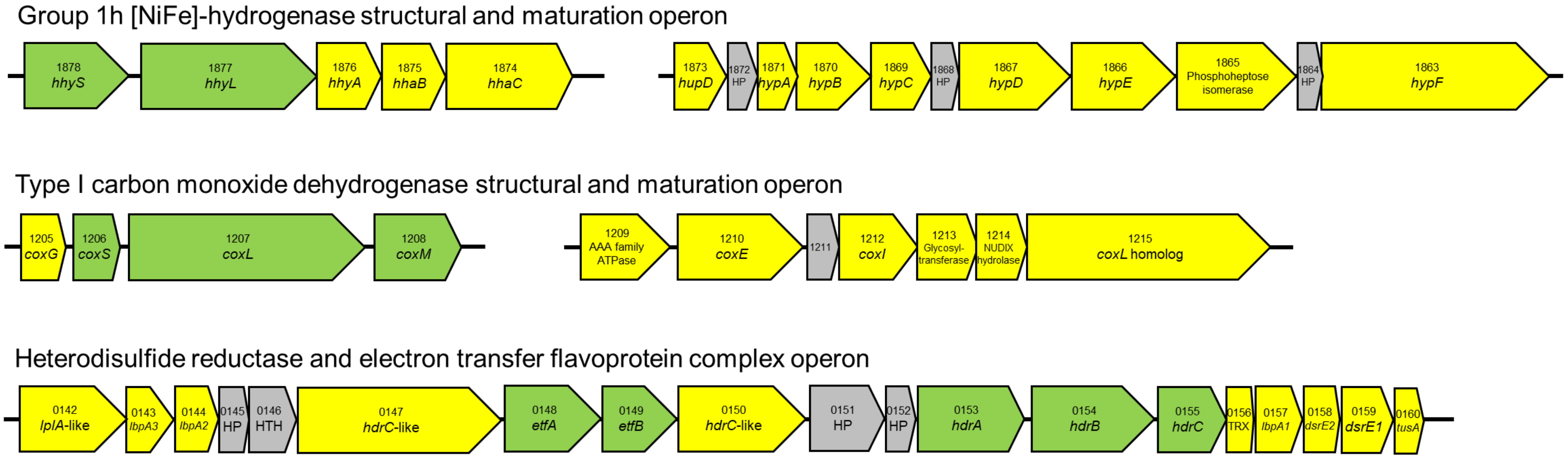

Figure S3. Structure of the putative operons encoding the group 1h [NiFe]-hydrogenase and type I carbon monoxide dehydrogenase in *Thermogemmatispora* sp. T81. Genes are differentially coloured depending on whether they encode structural subunits (green), accessory and maturation factors (yellow), or hypothetical proteins (grey).

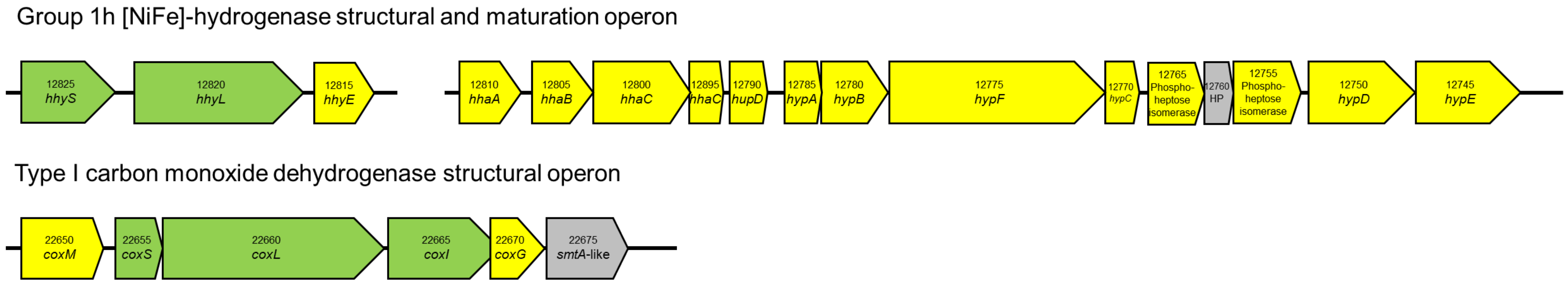

Figure S4. Phylogenetic tree showing the distribution of uptake respiratory hydrogenases in Chloroflexi genomes and metagenome-assembled genomes (MAGs). The genomes encode catalytic subunits of group 1a, group 1e, group 1f, group 1h, and group 2a [NiFe]-hydrogenases. Sequences are coloured by class, where blue = Ktenodobacteria, red = Chloroflexia, green = Dehalococcoidia, purple = Anaerolineae, and yellow = candidate class Ellin6529, with reference sequences in black. The tree was constructed using amino acid sequences of the hydrogenase large subunit using the neighbour-joining method and was bootstrapped with 100 replicates.

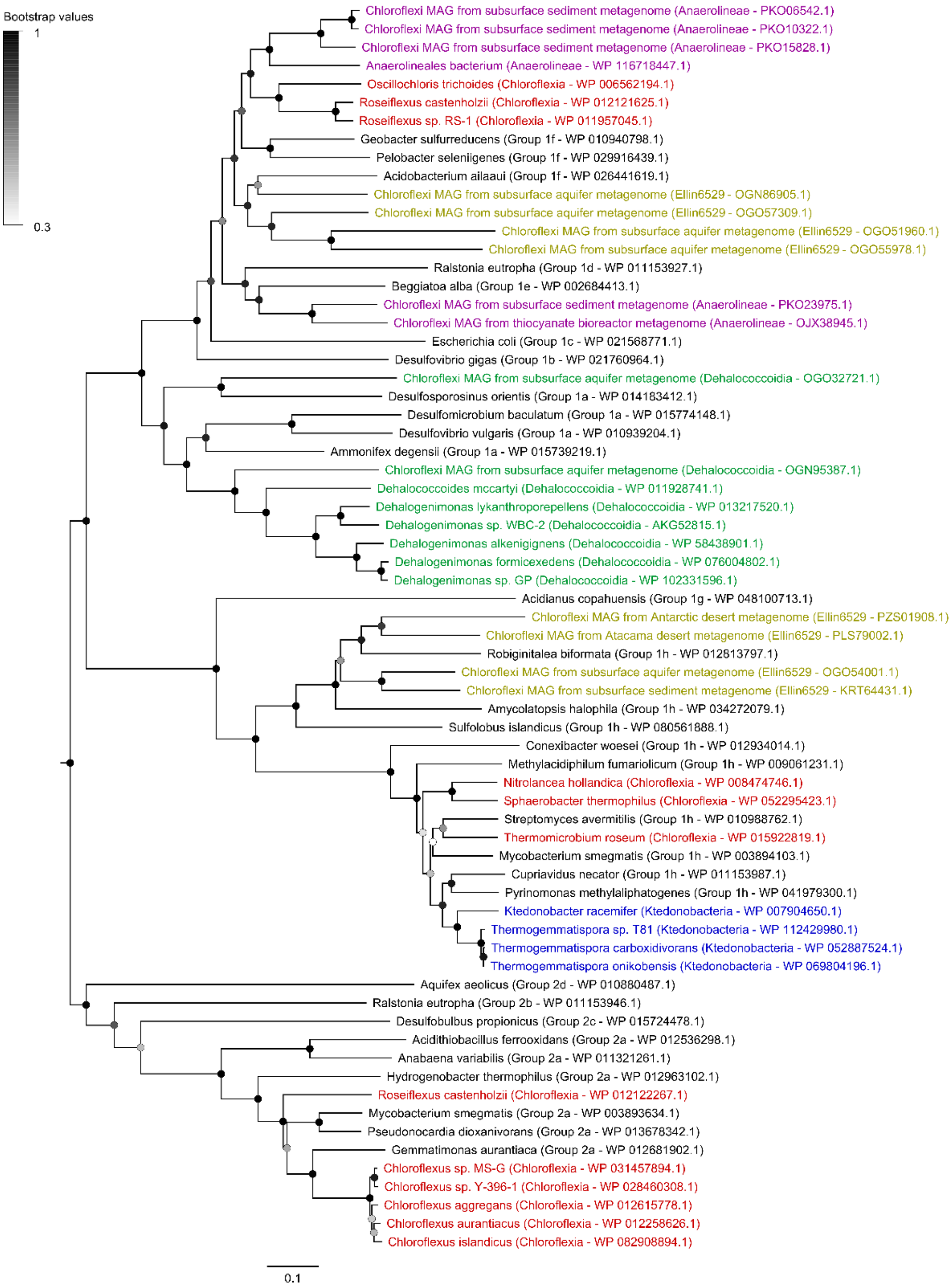

Figure S5. Phylogenetic tree showing the distribution of type I carbon monoxide dehydrogenases in Chloroflexi genomes and metagenome-assembled genomes (MAGs). Sequences are coloured by class, where blue = Ktenodobacteria, red = Chloroflexia, purple = Anaerolineae, and yellow = candidate class Ellin6529, with reference sequences in black. The tree was constructed using amino acid sequences of the carbon monoxide dehydrogenase large subunit using the neighbour-joining method and was bootstrapped with 100 replicates.

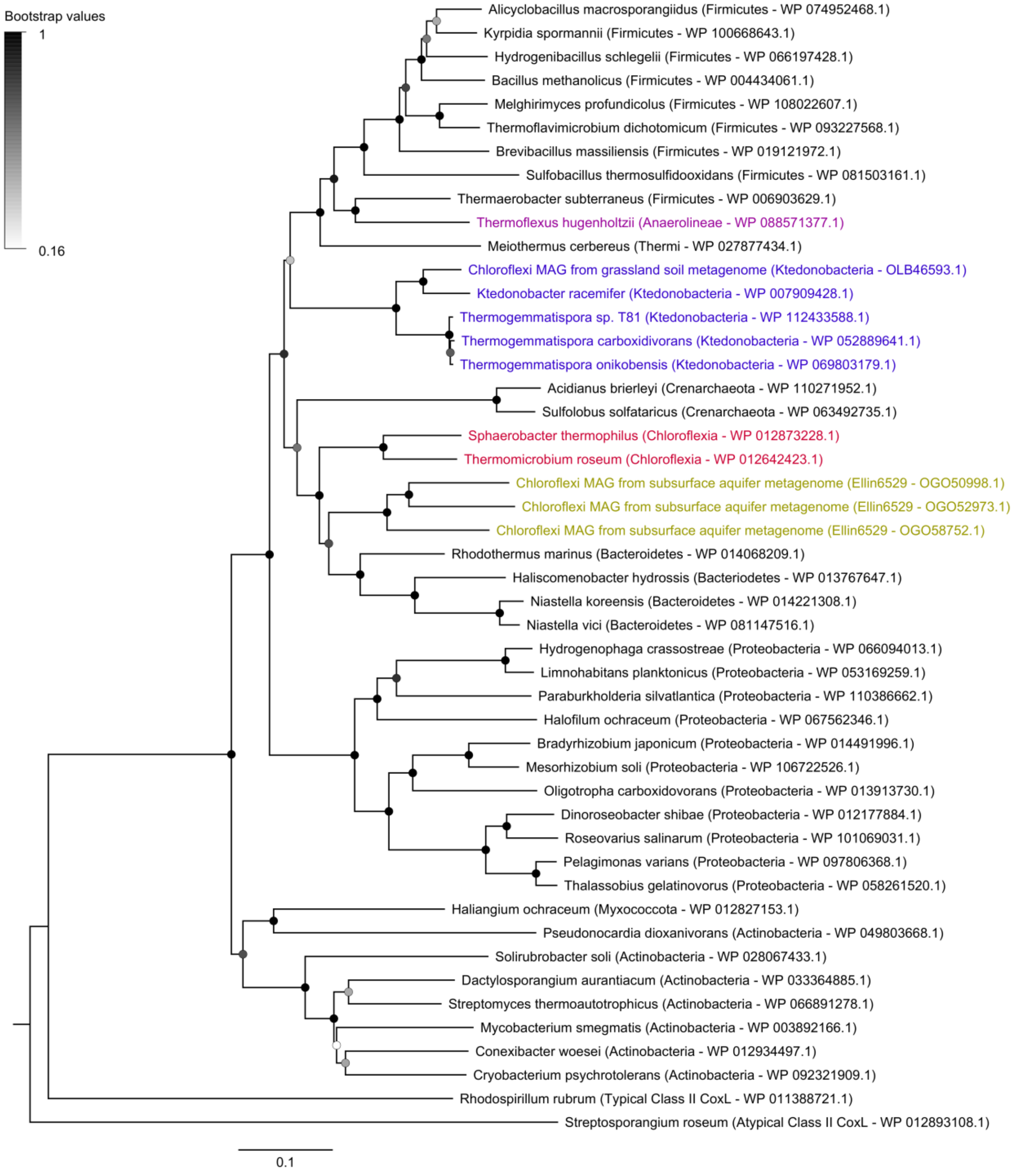

Supplement: Supplementary file 1 — Supplemental material [file 41396_2019_393_MOESM1_ESM.pdf]
